# Modulation of serotonin in the gut-liver neural axis ameliorates the fatty and fibrotic changes in non-alcoholic fatty liver

**DOI:** 10.1242/dmm.048922

**Published:** 2021-03-28

**Authors:** Masayoshi Ko, Kenya Kamimura, Takashi Owaki, Takuro Nagoya, Norihiro Sakai, Itsuo Nagayama, Yusuke Niwa, Osamu Shibata, Chiyumi Oda, Shinichi Morita, Atsushi Kimura, Ryosuke Inoue, Toru Setsu, Akira Sakamaki, Takeshi Yokoo, Shuji Terai

**Affiliations:** 1 Division of Gastroenterology and Hepatology, Graduate School of Medical and Dental Sciences, Niigata University, Niigata, 951-8510, Japan; 2 Department of General Medicine, Niigata University School of Medicine, Niigata, 951-8510, Japan

**Keywords:** Fatty liver, Autonomic neuron, Obesity, Diet, Hormone

## Abstract

The etiology of non-alcoholic fatty liver disease (NAFLD) consists of various factors, including neural signal pathways. However, the molecular mechanisms of the autonomic neural signals influencing NAFLD progression have not been elucidated. Therefore, we examined the involvement of the gut-liver neural axis in NAFLD development and tested the therapeutic effect of modulation of this axis in this study. To test the contribution of the gut-liver neural axis, we examined NAFLD progression with respect to body weight, hepatic steatosis, fibrosis, intestinal tight junction, microbiota and short-chain fatty acids in NAFLD models of choline-deficient defined L-amino-acid and high-fat diet-fed mice with or without blockades of autonomic nerves from the liver. Blockade of the neural signal from the liver to the gut in these NAFLD mice models ameliorated the progression of liver weight, hepatic steatosis and fibrosis by modulating serotonin expression in the small intestine. It was related to the severity of the liver pathology, the tight junction protein expression, microbiota diversity and short-chain fatty acids. These effects were reproduced by administrating serotonin antagonist, which ameliorated the NAFLD progression in the NAFLD mice models. Our study demonstrated that the gut-liver neural axis is involved in the etiologies of NAFLD progression and that serotonin expression through this signaling network is the key factor of this axis. Therefore, modulation of the gut-liver neural axis and serotonin antagonist ameliorates fatty and fibrotic changes in non-alcoholic fatty liver, and can be a potential therapeutic target of NAFLD.

This article has an associated First Person interview with the first author of the paper.

## Abstract (precis)

**Summary:** The gut-liver neural axis is involved in NAFLD progression and serotonin is the key factor in allowing this pathway to change the expression of tight junction molecules, microbiota diversity and short-chain fatty acids.

## INTRODUCTION

The number of non-alcoholic fatty liver disease (NAFLD) cases has been rising with the increasingly obese population. Globally, the reported prevalence of NAFLD is ∼30%, including 80%-90% of obese adults, 90% of patients with dyslipidemia and 40%-70% of obese children (Younossi et al., 2016). The etiology of the disease includes not only obesity and dyslipidemia but also impaired glucose tolerance, hypertension, insulin resistance, abnormal hormone secretion, genetic factors and gut-liver axis (Wiest et al., 2017; Tilg et al., 2020), and these factors have a complicated relationship (Drescher et al., 2019). The effect of the gut-liver axis has been focused on microbiota changes (Boursier et al., 2016; Hu et al., 2020), short-chain fatty acids (SCFAs) (De Vadder et al., 2014; Koopman et al., 2019) and tight junction (Miele et al., 2009; Gupta et al., 2020; Rahman et al., 2016), which are reportedly related to steatohepatitis, hepatic fibrosis (Loomba et al., 2017) and carcinogenesis (Yoshimoto et al., 2013) in NAFLD. However, as these factors are diverse and the pathologic network connecting these factors has not been elucidated, standard treatment methods for NAFLD have not been established. Recently, the multiple-organ network that runs through the autonomic neural pathway maintaining biological homeostasis, and is involved in various pathologies (Hayakawa et al., 2017; Kamiya et al., 2019; Imai et al., 2008; Izumi et al., 2018), has garnered attention. For example, the autonomic nervous activity contributes to the suppression of hyperglycemia during liver damage by regulating pancreas β cell activity (Imai et al., 2008), to the progression of cancers (Hayakawa et al., 2017; Kamiya et al., 2019), to liver regeneration upon severe liver damage (Izumi et al., 2018) and to inflammatory bowel diseases (Teratani et al., 2020). In addition, we recently demonstrated that the autonomic nervous system contributes to liver regeneration through signal transduction from the damaged liver to activate serotonin synthesis in the small intestine through the afferent sympathetic nerves and the brain (Inoue et al., 2018; Kamimura et al., 2018). Furthermore, we have reported the involvement of the gastric ghrelin activation that relayed the afferent autonomic neural signal from the stomach to the hypothalamus to release insulin-like growth factor-1 (IGF-1) from the liver to ameliorate its fatty change (Nagoya et al., 2020). According to previous reports and our findings, signal transmission of the autonomic nervous system from the liver is involved in various hepatic pathologies connecting various organs in the body (Inoue et al., 2018; Kamimura et al., 2018; Nagoya et al., 2020; Hurr et al., 2019). However, its role in NAFLD pathogenesis and progression remains elusive and has not been clarified in detail. Therefore, we hypothesized that the inter-organ autonomic neural connections are involved in NAFLD/non-alcoholic steatohepatitis (NASH) onset and progression. Of note, to our knowledge, no studies have directly shown the effect of gastrointestinal hormones on NAFLD pathology via neural signal transduction. We evaluated the effect of autonomic nervous circuits on the gut-liver axis in NAFLD/NASH progression by assessing hepatic steatosis, fibrosis, tight junction, microbiota, SCFA and serotonin, which is a gastrointestinal hormone as an effector, using choline-deficient defined L-amino-acid (CDAA) and high-fat diet (HFD)-fed NAFLD model mice.

## RESULTS

### Effect of autonomic nerve signal on body weight and liver weight in CDAA- and HFD-induced NAFLD mice models

**Fig. 1.**
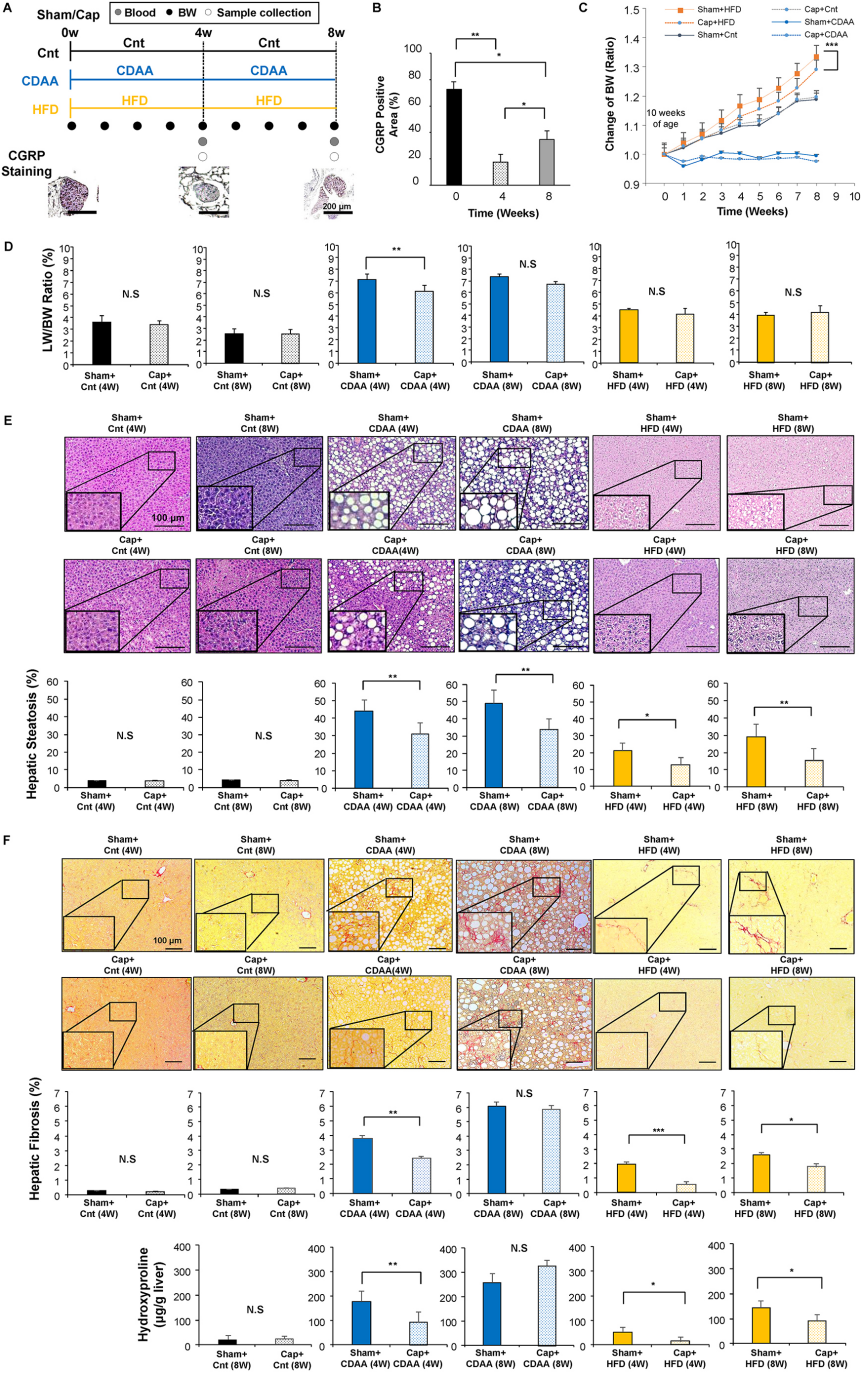
**Effect of autonomic neural signal transduction on NAFLD/NASH mice models.** (A) Experimental design. The mice were divided into 12 groups (*n*=6-8 mice per group): Sham plus Cnt (4W), Sham plus Cnt (8W), Sham followed by Cnt chow diet fed for 4 or 8 weeks; Cap plus Cnt (4W), Cap plus Cnt (8W). Direct topical application of capsaicin was used to deafferentate the afferent sympathetic nerve (Cap) followed by Cnt chow diet fed for 4 or 8 weeks; Sham plus CDAA (4W), Sham plus CDAA (8W), Sham followed by CDAA fed for 4 or 8 weeks; Cap plus CDAA (4W), Cap plus CDAA (8W), Cap followed by CDAA fed for 4 or 8 weeks; Sham plus HFD (4W), Sham plus HFD (8W), Sham followed by HFD fed for 4 or 8 weeks; Cap plus HFD (4W), Cap plus HFD (8W), Cap followed by HFD fed for 4 or 8 weeks. For each group, BW and LW were measured, and tissues and blood were collected at 4 and 8 weeks. (B) The efficacy of the neural blockade was confirmed by immunostaining of the nerves with anti-CGRP antibody. Three different sections from each of the five mice in the Cap plus Cnt groups were quantitatively analyzed for the CGRP^+^ cells using ImageJ software. Data are mean±s.d. (*n*=15 for each group) *P<0.05, **P<0.01, one-way ANOVA followed by Bonferroni's multiple comparison test. (C) Time-dependent change of BW. Data are mean±s.d. ***P<0.001 between Sham plus HFD and Cap plus HFD group, two-factor repeated measure ANOVA followed by Bonferroni's multiple comparison test. (D) LW/BW ratio. Representative images of H&E (E) and Sirius Red (F) staining of the livers. Scale bars: 100 μm. Five different sections from each of the five mice (*n*=25) in all groups were quantitatively analyzed for fatty infiltration and fibrotic tissue using ImageJ software. The bottom panels in F show hydroxyproline levels in the liver. Data are mean±s.d. *P<0.05; **P<0.01; ***P<0.001; N.S., not significant. Paired two-tailed Student's *t*-test.

Animal models were developed as described in Materials and Methods (Fig. 1A). The neural blockade showed the highest efficacy, with a 24% of decrease in neural signal activation, as evidenced by calcitonin gene-related peptide (CGRP, also known as Calca) staining at 4 weeks after capsaicin treatment (*P*<0.01), and slowly recovered to 48% of blockade in the following 4 weeks compared to that at 0 weeks (*P*<0.05) (Fig. 1B). To examine the effect of the visceral nerve on body weight (BW) and liver weight (LW) in the NAFLD mice models, the time-dependent changes in BW (Fig. 1C) and LW/BW ratio (Fig. 1D) were assessed. Although, as expected, the CDAA-fed mice model showed no observable BW gain, and the Cnt group showed no effect of visceral nerve blockade on BW gain [sham-operated group (Sham) plus control (Cnt), and capsaicin-treated group (Cap) plus Cnt], the HFD-fed group showed significant inhibition of BW gain with the afferent sympathetic nerve blockade (*P*<0.001), although food consumption was unchanged (Fig. S1A). LW/BW ratio showed no significant differences in the HFD-fed mice model with visceral nerve blockade, indicating that LW also inhibited its gain in developing NAFLD (Fig. 1D). In addition, the CDAA-fed mice model showed a lower LW/BW ratio within 4 weeks after denervation, suggesting that the gain in LW was significantly inhibited with denervation (*P*<0.01; Fig. 1D). These results suggest that neural signal transduction from the liver through the visceral nerve contribute to gain in LW and BW in HFD-fed mice and LW gain in CDAA-fed mice.

### Effect of autonomic nerve signal on hepatic steatosis in the CDAA- and HFD-induced NAFLD mice models

To determine whether signal transduction through the autonomic nerve system contributed to hepatic steatosis progression in the NAFLD mice models, we histologically assessed the amount of fatty tissue in the liver of CDAA- and HFD-fed mice with or without autonomic nerve blockade. The hepatic steatosis level in the liver was assessed quantitatively by the percentage of fat tissue area in the liver as reported previously (Nagoya et al., 2020) (Fig. 1E). Although no changes in fatty infiltration in the liver were seen with visceral nerve blockade and feeding with chow diet, the visceral nerve blockade contributed to suppress fatty infiltration at 4 weeks [Sham plus CDAA (4W) versus Cap plus CDAA (4W), *P*<0.01] and 8 weeks [Sham plus CDAA (8W) versus Cap plus CDAA (8W), *P*<0.01] of CDAA feeding (Fig. 1E). Similar results have been confirmed with HFD-fed mice showing inhibition of fatty infiltration in the liver after visceral nerve blockade followed by 4 weeks [Sham plus HFD (4W) versus Cap plus HFD (4W), *P*<0.05] and 8 weeks [Sham plus HFD (8W) versus Cap plus HFD (8W), *P*<0.01] of HFD feeding. Mild inhibition of macrophage infiltration (Fig. S1A) was observed; however, there were no changes in Foxp3 expression (Fig. S1B). These results suggest that the visceral nerve contributes to the accumulation of fatty tissue in the liver of the hepatic steatosis mice models, reflecting the gain in LW (Fig. 1C).

### Effect of autonomic nerve signal on hepatic fibrosis in CDAA- and HFD-induced NAFLD mice models

Based on the results regarding steatosis, we assessed the effect of the autonomic nervous signal transduction on hepatic fibrosis in the CDAA- and HFD-fed hepatic steatosis mice models. The amount of fibrotic tissue in the liver was assessed quantitatively by Sirius Red staining and the level of hydroxyproline level in the liver tissue (Fig. 1F). Although no difference in fibrosis level was seen with the blockade of the autonomic neural signal transduction from the liver in mice treated with a chow diet [Sham plus Cnt (4W) versus Cap plus Cnt (4W), NS; Sham plus Cnt (8W) versus Cap plus Cnt (8W), NS] and a lower detection limit for hydroxyproline, the blockade contributed to suppress hepatic fibrosis after 4 weeks of CDAA feeding [histologic analysis: Sham plus CDAA (4W) versus Cap plus CDAA (4W), *P*<0.01; hydroxyproline: Sham plus CDAA (4W) versus Cap plus CDAA (4W), *P*<0.01; Fig. 1F]. With the significant amount of fibrotic tissue after 8 weeks of CDAA feeding, no effect of neural blockade was noted. Similar results have been confirmed with HFD-fed mice showing inhibition of hepatic fibrosis in the liver after visceral nerve blockade followed by 4 weeks [histologic analysis: Sham plus HFD (4W) versus Cap plus HFD (4W), *P*<0.001; hydroxyproline: Sham plus HFD (4W) versus Cap plus HFD (4W), *P*<0.05; Fig. 1F] and 8 weeks (histological analysis: Sham plus HFD (8W) versus Cap plus HFD (8W), *P*<0.05; hydroxyproline: Sham plus HFD (8W) versus Cap plus HFD (8W), *P*<0.05; Fig. 1F] of HFD feeding. These results suggested that the visceral nerve contributes to the progression of steatohepatitis in CDAA- and HFD-fed mice NASH models.

To determine the mechanisms of the anti-steatosis and anti-fibrotic changes in the liver after visceral nerve blockade, we examined changes in tight junctions of the small intestine, intestinal bacterial flora and SCFAs, which are reportedly related to NAFLD and NASH development (Koopman et al., 2019; Rahman et al., 2016).

### Effect of autonomic nerve signal on the tight junction in the small intestine in CDAA- and HFD-induced NAFLD mice models

**Fig. 2.**
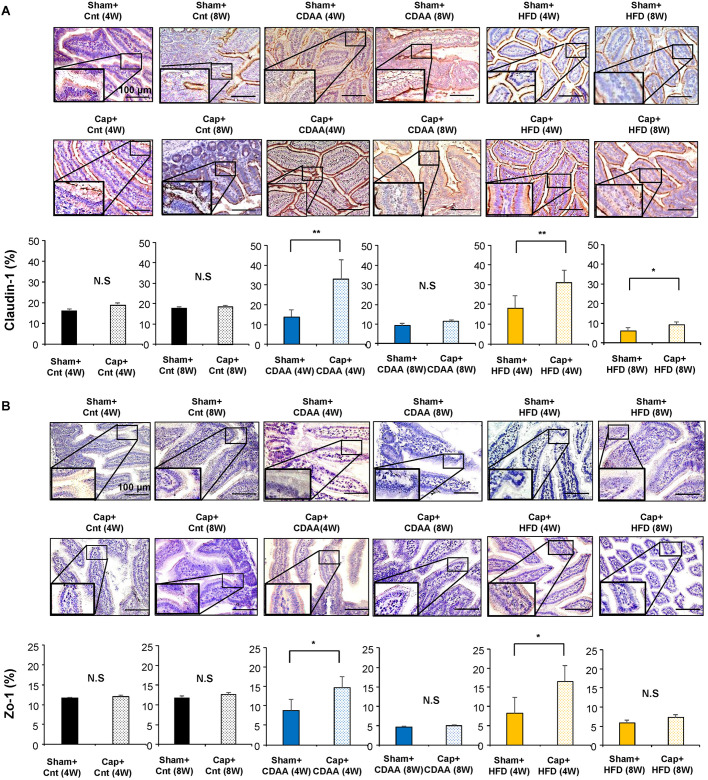
**Effect of autonomic neural signal transduction on the tight junction in the small intestine of NAFLD/NASH mice models.** (A,B) Representative images of Claudin-1 (A) and Zo-1 (B) staining of the small intestine of mice groups. Five different sections from each of the five mice (*n*=25) in all groups were quantitatively analyzed for the positively stained area using ImageJ software. Data are mean±s.d. *P<0.05; **P<0.01; N.S., not significant. Paired two-tailed Student's *t*-test. Scale bars: 100 μm.

To examine the effect of the visceral nerve signal on the tight junction in NAFLD, levels of Claudin-1 and Zo-1 expression in the small intestine of NAFLD mice models with or without visceral nerve blockade were analyzed quantitatively by immunostaining (Fig. 2A,B). Although no changes in Claudin-1 and Zo-1 (also known as Tjp1) expression in the small intestine were seen with visceral nerve blockade feeding with a chow diet [Sham plus Cnt (4W) versus Cap plus Cnt (4W), NS; Sham plus Cnt (8W) versus Cap plus Cnt (8W), NS (Fig. 2A); Sham plus Cnt (4W) versus Cap plus Cnt (4W), NS; Sham plus Cnt (8W) versus Cap plus Cnt (8W), not significant (Fig. 2B), respectively] the visceral nerve blockade contributed in activating expression of Claudin-1 [Sham plus CDAA (4W) versus Cap plus CDAA (4W), *P*<0.01; Fig. 2A] and Zo-1 [Sham plus CDAA (4W) versus Cap plus CDAA (4W), *P*<0.01; Fig. 2B] after 4 weeks of CDAA feeding. This effect recovered in the following 4 weeks in Claudin-1 [Sham plus CDAA (8W) versus Cap plus CDAA (8W), NS; Fig. 2A] and Zo-1 (Sham plus CDAA (8W) versus Cap plus CDAA (8W), not significant; Fig. 2B) expressions. A similar effect also was seen in HFD-fed mice for 4 weeks on the expression of Claudin-1 [Sham plus HFD (4W) versus Cap plus HFD (4W), *P*<0.01; Fig. 2A] and Zo-1 [Sham plus HFD (4W) versus Cap plus HFD (4W), *P*<0.05; Fig. 2B]. This effect also slowly recovered in the following 4 weeks, which is similar to the CDAA NAFLD model [Sham plus HFD (8W) versus Cap plus HFD (8W), *P*<0.05 (Fig. 2A); Sham plus HFD (8W) versus Cap plus HFD (8W), not significant (Fig. 2B)]. These results suggest that signal transduction through the visceral nerve contributes in weakening intestinal tight junction expression in the hepatic steatosis mice models.

### Effect of autonomic nerve signal on the microbiota and SCFA in CDAA- and HFD-induced NAFLD mice models

**Fig. 3.**
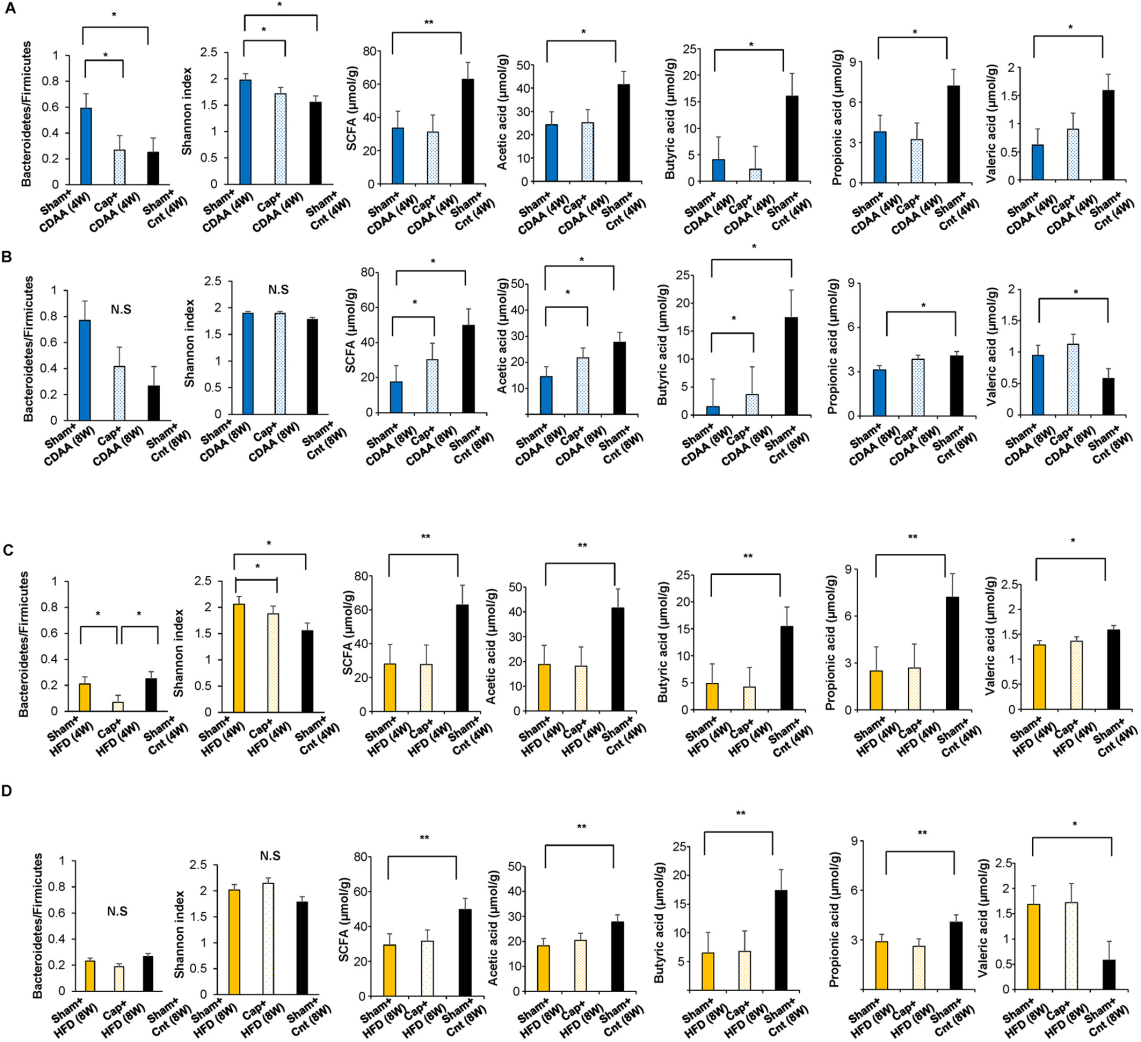
**Effect of autonomic neural signal transduction on the microbiota and SCFA of NAFLD/NASH mice models.** (A-D) The *Bacteroidetes*/*Firmicutes* ratio, Shannon index analysis of the gut microbiota, changes of total amount of SCFAs, acetic acid, butyric acid, propionic acid and valeric acid. Mice were fed with CDAA (4W) (A), CDAA (8W) (B), HFD (4W) (C) and HFD (8W) (D). Each of the six mice in all groups were quantitatively analyzed for the structure of the gut microbiota (using T-RFLP analysis) and for SCFAs. Data are mean±s.d. *P<0.05; **P<0.01; N.S., not significant (one-way ANOVA followed by Bonferroni's multiple comparison test).

As tight junction protein expression is related to the intestinal bacterial flora (Nakata et al., 2017) and to NAFLD and NASH development (Miele et al., 2009), we examined and analyzed by terminal restriction fragment length polymorphism (T-RFLP), with or without visceral nerve blockade, the changes in intestinal bacterial flora in these NAFLD mice models (Fig. 3). Although the CDAA diet significantly increased the *Bacteroidetes*/*Firmicutes* ratio compared to that of the control diet-fed mice (Fig. 3A), the visceral nerve blockade caused by capsaicin in the CDAA-fed mice resulted in a decreased ratio after 4 weeks [Sham plus CDAA (4W) versus Cap plus CDAA (4W), *P*<0.05; Fig. 3A] (Fig. S2). A similar tendency was seen after 8 weeks [Sham plus CDAA (8W) versus Cap plus CDAA (8W); Fig. 3B]. Similar but milder results have been confirmed with HFD-fed mice after 4 weeks of neural blockade [Sham plus HFD (4W) versus Cap plus HFD (4W), *P*<0.05; Fig. 3C], but no differences were seen 8 weeks after the blockade (Fig. S2).

The microbial diversity in the groups was assessed by the Shannon index analysis (Fig. 3). The Shannon index revealed that the CDAA diet significantly affected the diversity compared to the sham-operated control diet-fed mice (1.55±0.15) and that the visceral nerve blockade showed recovery of the intestinal flora [Sham plus CDAA (4W) versus Cap plus CDAA (4W), *P*<0.05; Fig. 3A]. The difference became milder in the following 4 weeks [Sham plus CDAA (8W) versus Cap plus CDAA (8W), not significant; Fig. 3B]. Similar results have been confirmed with HFD-fed mice [Sham plus HFD (4W) versus Cap plus HFD (4W), *P*<0.05 (Fig. 3C); Sham plus HFD (8W) versus Cap plus HFD (8W), not significant (Fig. 3D)]. These results indicate that the NAFLD mice models fed with CDAA and HFD show a partial relationship to the changes in the intestinal bacterial flora and that the blockade of the neural signal transduction from the NAFLD liver may have contributed to the diversity and inhibited progression of NAFLD.

Next, the changes in SCFAs, the product of the intestinal bacterial flora, were assessed, as it they are significantly related to the condition of the intestinal bacterial conditions and NASH. The total SCFAs, acetic acid, butyric acid, propionic acid and valeric acid were assessed in the feces of mice models. Although total SCFAs decreased when fed with CDAA (Fig. 3A,B) and HFD (Fig. 3C,D), the blockade of the neural signal transduction recovered SCFA levels [Sham plus CDAA (8W) versus Cap plus CDAA (8W), *P*<0.05], acetic acid [Sham plus CDAA (8W) versus Cap plus CDAA (8W), *P*<0.05], and butyric acid [Sham plus CDAA (8W) versus Cap pus CDAA (8W), *P*<0.05; Fig. 3B] 8 weeks after neural blockade in the CDAA-fed mice. No significant differences in SCFAs in HFD-fed mice were noted (Fig. 3C,D). The time lag of changes seen in bacterial diversity and amount of SCFAs is understandable as SCFAs are the product of the bacteria. These results suggest that changes in the intestinal bacterial flora in CDAA- and HFD-fed mice depend on the autonomic nervous signals from the liver, and that SCFAs are partly related to this phenomenon (Fig. 3).

As intestinal inflammation also has been related to NASH development (Mikami et al., 2014), the levels of F4/80^+^ macrophages and Foxp3^+^ regulatory T cells in the small intestine were analyzed quantitatively by immunostaining (Fig. S3). The Foxp3^+^ and F4/80^+^ cells in the small intestine of control diet-, CDAA- and HFD-fed mice showed low levels of positively stained cells, and no differences were seen, indicating that the F4/80^+^ macrophages were not involved in NASH development with CDAA and HFD feeding.

### Visceral nerve blockade suppressed serotonin expression in NAFLD mice

**Fig. 4.**
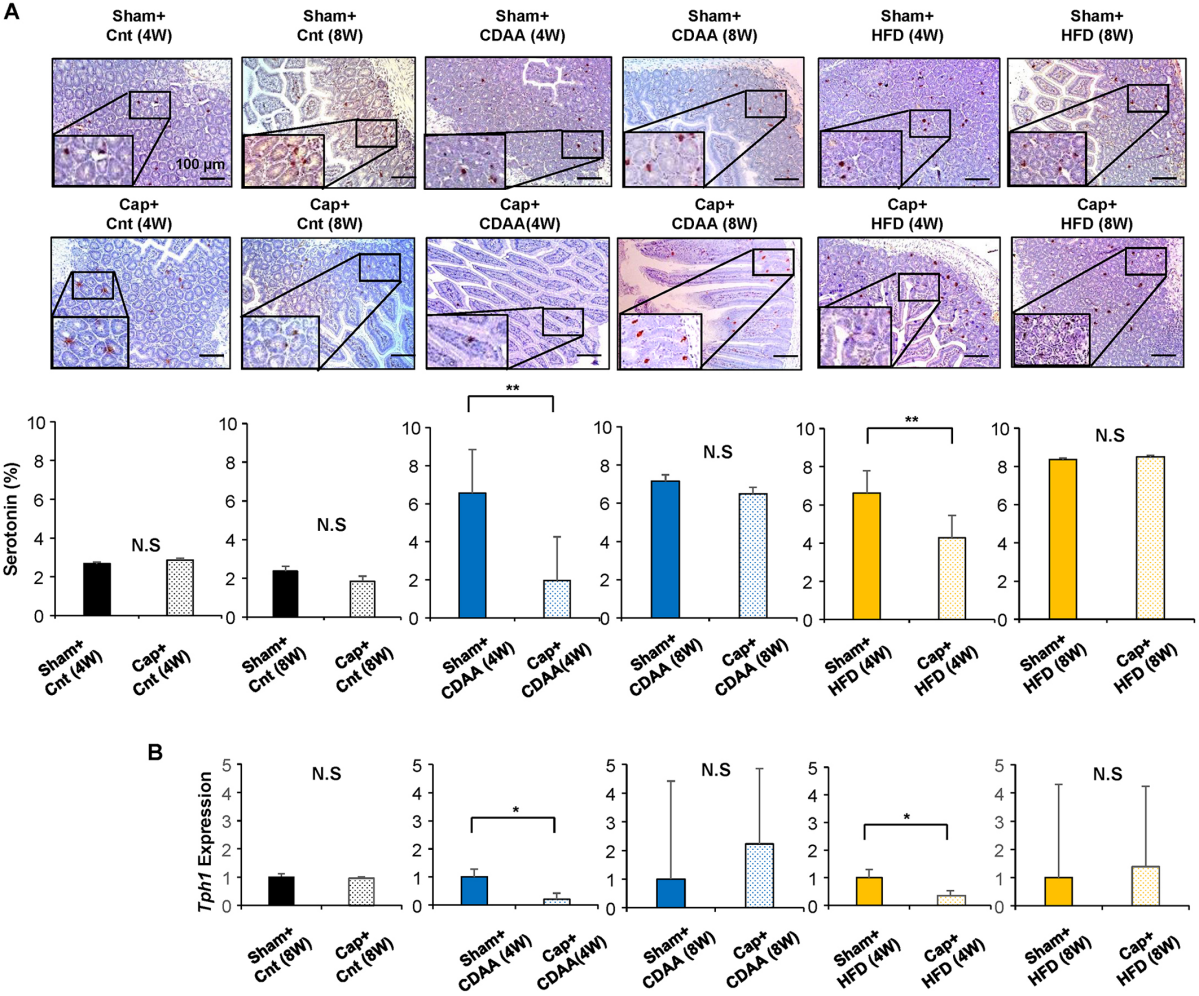
**Expression of serotonin in the small intestine of NAFLD/NASH mice models.** (A) Representative images of serotonin expression in the small intestine of the model mice. Five different sections from each of the five mice (*n*=25) in all groups were quantitatively analyzed for the positively stained area using ImageJ software. (B) Expression of tryptophan hydroxylase 1 in the small intestine. *Gapdh* was used as an internal control. Data are mean±s.d. *P<0.05; **P<0.01; N.S., not significant (paired two-tailed Student's *t*-test).

Because the expression of serotonin, a gastrointestinal hormone, is significantly related to hepatic regeneration upon liver injury, intestinal tight junctions (Haub et al., 2010, 2011) and intestinal bacterial flora (Ge et al., 2018; Yano et al., 2015; Lyte et al., 2020), and is inhibited by the autonomic nervous signal transduction blockade, particularly afferent sympathetic nerves from the injured liver (Inoue et al., 2018; Nagoya et al., 2020), we examined serotonin expression in the small intestine as the effector of CDAA- and HFD-induced NAFLD in mice liver. The levels of serotonin expression in the enterochromaffin cells in the small intestine were quantitatively analyzed by immunostaining in CDAA- and HFD-fed mice models with or without the visceral nervous signal blockade from the liver (Fig. 4A). Although no changes in serotonin expression in the small intestine were seen with visceral nerve blockade feeding with the chow diet [Sham plus Cnt (4W) versus Cap plus Cnt (4W), not significant; Sham plus Cnt (8W) versus Cap plus Cnt (8W), not significant; Fig. 4A], CDAA- and HFD-fed mice showed increased serotonin expression in the small intestine, and the visceral nerve blockade contributed in suppressing the serotonin level in the small intestine after 4 weeks of CDAA feeding [Sham plus CDAA (4W) versus Cap plus CDAA (4W), *P*<0.01; Fig. 4A] and HFD [Sham plus HFD (4W) versus Cap plus HFD (4W), *P*<0.01; Fig. 4A]. The mRNA expression level of *Tph1*, an enzyme catalyzing serotonin, also showed that the visceral nerve blockade inhibits its expression 4 weeks after CDAA and HFD feeding (Fig. 4B, *P*<0.05). These results suggest that the effect of the visceral nerve blockade on the inhibition of the steatosis may be based partly on decreased serotonin expression in the enterochromaffin cells in the small intestine. Although the serum biochemical analyses of the CDAA-fed mice group showed no significant changes (Table 1), inhibition of hepatobiliary enzymes and triglycerides (TG) and NH_3_ in the HFD-induced NAFLD mice was observed (Table 1).

**Table 1.**
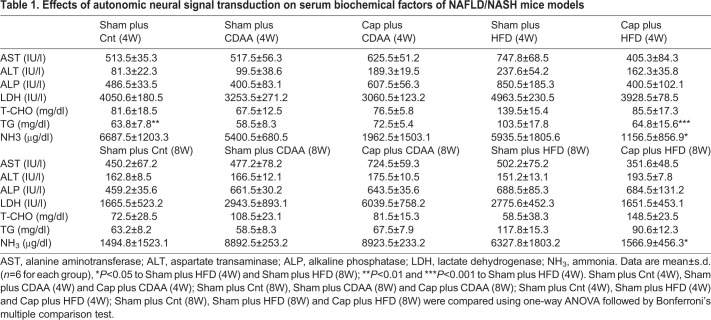
**Effects of autonomic neural signal transduction on serum biochemical factors of NAFLD/NASH mice models**

**Fig. 5.**
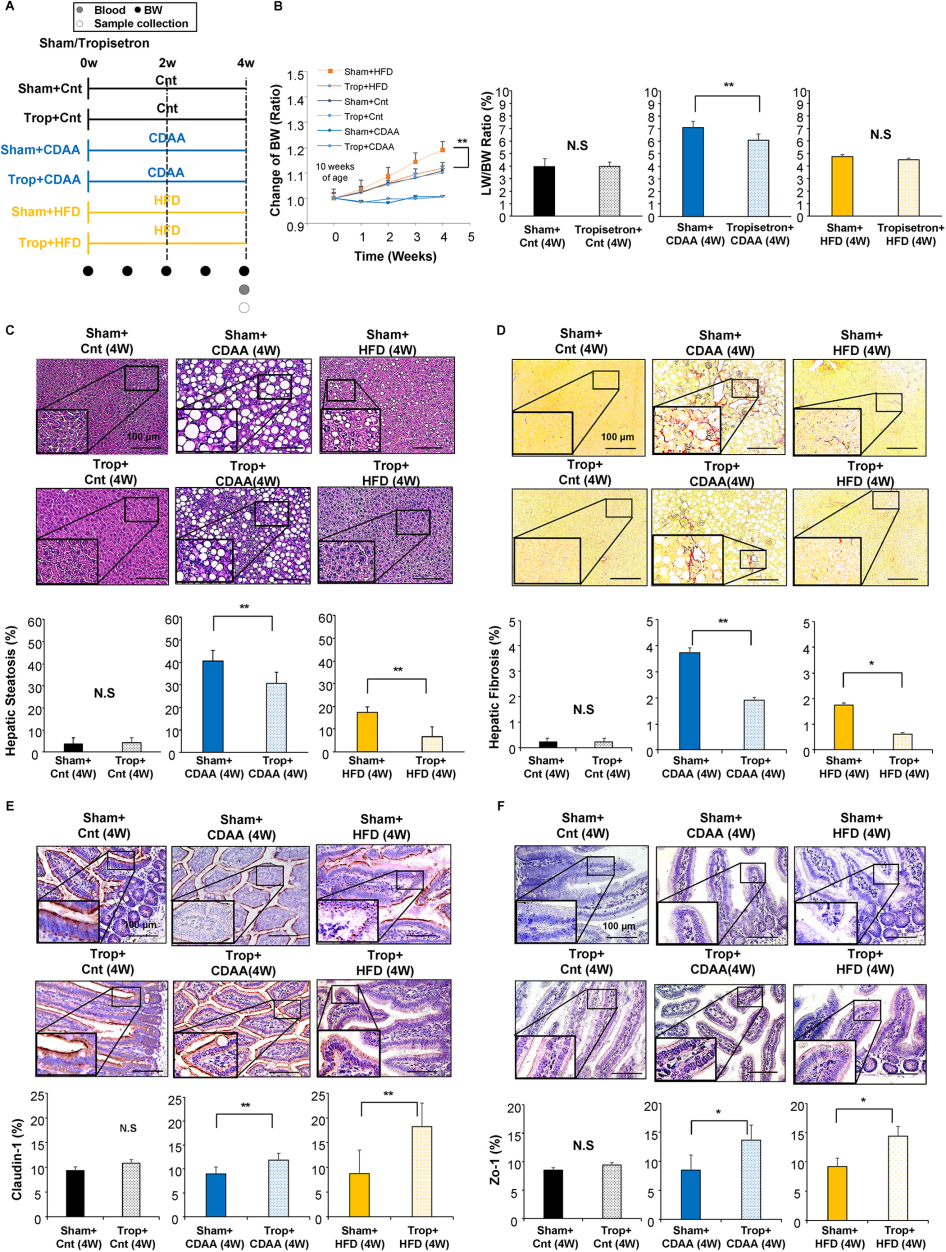
**Effect of serotonin antagonist on NAFLD/NASH mice models.** (A) Experimental design. The mice were divided into six groups (*n*=5 mice for each group). Trop, tropisetron. (B) Time-dependent change of BW. LW/BW ratio. (C-F) Representative images of H&E (C) and Sirius Red (D) staining of the livers, and Claudin-1 (E) and Zo-1 (F) staining of the small intestine of mice groups. Five different sections from each of the five mice (*n*=25) in all groups were quantitatively analyzed for fatty infiltration, fibrotic tissue and positively stained area using ImageJ software. Data are mean±s.d. *P<0.05; **P<0.01; N.S., not significant. Two-factor repeated measure ANOVA followed by Bonferroni's multiple comparison test (B); Paired two-tailed Student's *t*-test (C-F). Scale bars: 100 μm.

To demonstrate the role of serotonin on the etiology of steatosis and fibrosis via the autonomic nervous signal transduction in CDAA- and HFD-induced NAFLD, tropisetron, a serotonin antagonist that blocks the 5-hydroxytryptamine3 (5-HT3) receptor expressed mainly in the intestine (Amini-Khoei et al., 2016), was used to assess its effect on hepatic steatosis, fibrosis and the tight junction of the small intestine (Fig. 5). Fig. 5A shows the design of the study.

Although the Cnt and CDAA-fed group showed no observable BW gain with the administration of tropisetron, the HFD-fed group showed significant inhibition of BW gain with tropisetron (Fig. 5B; *P*<0.01), although the food consumption showed no difference, consistent with the results obtained with the visceral nervous blockade (Fig. 1). The LW/BW ratio showed no significant differences in the HFD-fed mice model with visceral nerve blockade, indicating that LW also inhibited its gain (Fig. 5B). In addition, the CDAA-fed mice model showed lower LW/BW ratios within 4 weeks after denervation, suggesting that the gain in LW was inhibited significantly with denervation (Fig. 5B, *P*<0.01), also consistent with the results obtained with visceral nerve blockade.

Inhibition of the hepatic steatosis also was confirmed in these CDAA- and HFD-fed mice models when treated with tropisetron for 4 weeks, similar to the results obtained with visceral nerve blockade [CDAA (4W) versus tropisetron plus CDAA (4W), *P*<0.01; HFD (4W) versus tropisetron plus HFD (4W), *P*<0.01; Fig. 5C]. Hepatic fibrosis also was inhibited with tropisetron [CDAA (4W) versus tropisetron plus CDAA (4W), *P*<0.01; HFD (4W) versus tropisetron plus HFD (4W), *P*<0.05; Fig. 5D]. Expression of Claudin-1 and Zo-1 proteins in the CDAA- and HFD-fed mice was increased when treated with tropisetron [Claudin-1: CDAA (4W) versus tropisetron plus CDAA (4W), *P*<0.01; HFD (4W) versus tropisetron plus HFD (4W), *P*<0.01 (Fig. 5E); Zo-1 CDAA (4W) versus tropisetron plus CDAA (4W), *P*<0.05; HFD (4W) versus tropisetron plus HFD (4W), *P*<0.05 (Fig. 5F)]. Overall, the results obtained with tropisetron administration in the CDAA- and HFD-fed mice were similar to the effect of capsaicin treatment on tight junction protein expression. These results suggested that the effect of the visceral nerve on NAFLD progression in CDAA- and HFD-fed mice models was partly due to the effect of serotonin.

## DISCUSSION

Our study demonstrated that autonomic nervous signal transduction from the liver contributed to the development of NAFLD/NASH in HFD- and CDAA-fed mice through the activation of serotonin expression. Therefore, autonomic nervous signal blockade from the liver and serotonin antagonist administration showed amelioration of NAFLD/NASH. These results supported our hypothesis that the autonomic nervous signals from the liver to the gut are involved in the pathogenesis of NAFLD/NASH onset and progression through neural signal transduction. Furthermore, serotonin has been shown to be an effector of this gut-liver neural axis in NAFLD/NASH pathology by modifying the expression of tight junction proteins, the composition of the microbiota and SCFAs.

The involvement of the autonomic nervous system has been reported to be a key factor connecting the various organs in the body (Hayakawa et al., 2017; Kamiya et al., 2019; Teratani et al., 2020). In addition, activating the autonomic nerves is known to change the gastrointestinal microbiota and the composition of intestinal microbiota (Bonaz et al., 2018). Butyric acid is involved in communication between the gastrointestinal microbiota and the brain (Raybould, 2010). For liver diseases, the neural signal has been reported to be related to the increased pancreatic β cells to regulate blood sugar after hepatectomy to maintain biological homeostasis (Imai et al., 2008), activation of the parasympathetic nervous system and FoxM1 pathway for liver regeneration (Izumi et al., 2018), energy metabolism of the liver (Conde-Sieira et al., 2020) and fatty change of the liver in the obese mice model (Hurr et al., 2019). Therefore, it is reasonable to hypothesize that the pathology of NAFLD/NASH involves autonomic nervous signal transduction from the liver. Our results support the evidence that serotonin is a key factor involved in this pathway (Figs 4, 6). Serotonin is a monoamine neurotransmitter, 90% of which has been found in the gastrointestinal tract, synthesized from tryptophan, and secreted from chromaffin cells of the small intestine (Berger et al., 2009). Serotonin is also known to have numerous functions, such as smooth muscle contraction of blood vessels and bronchi, acceleration of peristaltic movement (Berger et al., 2009), respiratory rhythm (Cinelli et al., 2020), regulation of β cells in the pancreas (Berger et al., 2009; Li et al., 2000), liver regeneration (Inoue et al., 2018; Kamimura et al., 2018), liver fibrosis (Kyritsi et al., 2020), protection against intestinal ischemia (Tackett et al., 2019) and food-seeking behavior (He et al., 2020), and its reuptake inhibitors are used as an antidepressant in relation to gastrointestinal symptoms (Salisbury et al., 2020). In addition, serotonin is reportedly involved in the fibrosis of NAFLD (Atallah et al., 2018), the modification of lipid metabolisms (Namkung et al., 2018), the activation of signal from serotonin receptor on the hepatocytes (Choi et al., 2018), and the modulation of autophagy and Notch signal (Martin et al., 2020). Interestingly, Haub et al. (2011) reported that the blockade of the serotonin receptor HTR3, which is the receptor expressed in the intestine, modified the strength of the tight junction and improved the obesity-associated fatty liver in mice. In addition, Choi et al. (2018) recently reported that the selective inhibition of HTR2A, which is the receptor expressed in the central nervous system, prevented HFD-induced hepatic steatosis. These previous studies supported our findings that serotonin may play a key role in the NAFLD/NASH mice models; however, its relationship with the liver, i.e. to signal the activation of serotonin expression, has not been clarified to date. Therefore, our results first demonstrate the effect of the interorgan communication in NAFLD pathogenesis through the autonomic neural relay from the liver and the role of serotonin in this axis.

In addition, although changes in the tight junction with serotonin antagonist administration have been reported in obese mice (Haub et al., 2010, 2011) and the microbiota affect serotonin synthesis in the small intestine (Ge et al., 2018; Yano et al., 2015; Lyte et al., 2020), our results demonstrate that serotonin expression is associated with the expression of tight junction proteins (Fig. 2), composition of the microbiota (Fig. 3) and part of SCFA (Fig. 3) with time-dependent histologic changes in the NAFLD/NASH (Kwon et al., 2019; Vincent et al., 2018) and LW and BW gain (Figs 1, 5). Although the neuron-immune cell crosstalk has been reported in the pathologies of gastrointestinal disease, no significant changes were seen in the inflammatory changes in the intestine (Fig. S3). These results also support the finding that the autonomic nervous signal effect in the development of NAFLD/NASH is related partly to serotonin.

The inhibitory effect of the neural blockade on NAFLD/NASH development in the histology, tight junction protein expression, microbiota and SCFA composition, and serotonin expression was highest at 4 weeks of blockade. This effect gradually returned in the following 4 weeks due to the decreased blocking effect of capsaicin and recovery of the neural signal transduction, as evidenced by the recovery of the stainability with CGRP (Fig. 1B). This result indicates that a continuous signal through the autonomic nervous system contributes to NAFLD/NASH development. In addition, continuous blockade of the signal could provide a longer effect on preventing NAFLD progression. Importantly, these phenomena have been confirmed by administering tropisetron, the 5HTR3 antagonist, to the mice models (Fig. 5). The limitation of our study was that results were evident in the mice models, and effects of serotonin on NASH development should be assessed further as it partly affected the infiltration of inflammatory cells (Fig. S1B,C). In addition, lipopolysaccharides/bacterial DNA measurement in blood and SCFA in fecal samples and blood will be helpful to examine the direct relationship between serotonin and the intestinal barrier function, as well as the effects of serotonin antagonists. As tropisetron is approved as an anti-emetic medicine, further study focusing on human tissue samples is necessary for the translational potential of the study.

In summary, our study demonstrates that the autonomic nervous signal transduction of the gut-liver axis is involved in the development of NAFLD and that serotonin expression through this signaling network is the key factor of this axis. The mechanisms include management of serotonin expression, which is associated with the expression of tight junction proteins, microbiota composition and SCFAs. The results suggest that the management of serotonin signal in a serotonin receptor-dependent manner could be a novel therapeutic option for NAFLD/NASH.

## MATERIALS AND METHODS

### Animals

All animal experiments were approved by and conducted in full compliance with the regulations of the institutional animal care and use committee at Niigata University, Niigata, Japan (SA00568). Male C57BL/6JJcl mice (*n*=120, 8 weeks old and 20-25 g; purchased from Charles River Japan, Yokohama, Kanagawa, Japan) were housed in standard conditions at a temperature of 20°C-23°C and humidity of 45%-55% in specific pathogen-free facilities. Three mice were housed in a cage, their body weight and food volume were measured daily, and their consumption was calculated based on the difference of the remaining food volume in the cage.

### Development of animal models

Mice were first divided into Sham and a Cap groups at 10 weeks of age. For the Cap group, the direct topical application of capsaicin (Wako Pure Chemical Industries, Osaka, Japan) dissolved in olive oil (50 mg/ml) was used to deafferent the afferent sympathetic fibers from the liver. For this deafferentation, the celiac artery was exposed by laparotomy incision and wrapped in gauze immersed with (Cap) or without (Sham) capsaicin for 30 min as described previously (Imai et al., 2008; Inoue et al., 2018; Nagoya et al., 2020). Although this method has been reported to not affect other nerves, including the vagus nerves (Inoue et al., 2018; Hurr et al., 2019; Warne et al., 2007), both groups were given the same food allotments, and consumption was measured to demonstrate that capsaicin has no effect on hunger and food intake (Fig. S1A). Next, these mice groups were divided further into six groups according to diet: Cnt, mice fed with standard diet for 4 weeks [Sham plus Cnt (4W), Cap plus Cnt (4W)] or 8 weeks [Sham plus Cnt (8W), Cap plus Cnt (8W)]; mice fed with CDAA containing 0.1% of methionine, 62% kcal fat, 18% kcal protein and 20% kcal carbohydrate (A06071302; Research Diets, New Brunswick, NJ, USA) for 4 weeks [Sham plus CDAA (4W), Cap plus CDAA (4W)] or 8 weeks [Sham plus CDAA (8W), Cap plus CDAA (8W)] weeks; and mice fed with HFD containing 60% kcal fat, 20% kcal protein and 20% kcal carbohydrate (D12492; Research Diets) for 4 weeks [Sham plus HFD (4W), Cap+HFD (4W)] or 8 weeks [Sham plus HFD (8W), Cap plus HFD (8W)] (Fig. 1A).

To assess the effect of tropisetron (C17H20N2O2; Tokyo Kasei, Tokyo, Japan), a serotonin antagonist, on mice fed with CDAA and HFD for 4 weeks, tropisetron was administered to mice as reported previously (Haub et al., 2010), adjusting the concentration in the drinking water according to BW so that the animals could receive ∼0.2 mg/kg tropisetron per day orally. The volume of food consumption was measured with mice housed in-group every 24 h, as the difference in the amount of food in the cage providing an average intake/mouse.

### Histological analysis

Tissue samples for immunohistochemical staining were collected at 4 and 8 weeks after the procedures in each group and fixed in 10% formalin upon tissue collection before embedding in paraffin. Five different sections from each lobe of the liver and from the small intestine (10 μm) were obtained from each of the five mice, and standard Hematoxylin and Eosin (H&E) staining, Sirius Red staining and immunohistochemistry were performed. For the small intestine, the full length of the intestine was obtained, and five sections were collected with even intervals for all samples to ensure reliability and reproducibility. Adipose tissue in the liver and fibrotic tissue was detected by H&E and Sirius Red staining, respectively. For immunohistochemical stainings, anti-serotonin monoclonal antibody (M0758, DAKO, CA, USA, 1:50) anti-Claudin-1 antibody (ab15098, Abcam, 1:100) and anti-Zo-1 antibody (bs-1329R, Bioss, MA, USA, 1:200) with a Vectastain Elite ABC mouse IgG kit (PK-6102, Vector Laboratories, CA, USA) and diaminobenzidine (DAB) chromogen tablets (Muto Pure Chemicals, Tokyo, Japan), and anti-CGRP monoclonal antibody (ab81887, Abcam, 1:100), anti-F4/80 antibody (ab111101, Abcam, 1:100) and anti-forkhead box P3 (Foxp3) antibody (GTX107737, Genetex, CA, USA, 1:250) with a Vectastain Elite ABC rabbit IgG kit (PK-6101, Vector Laboratories) and DAB chromogen tablets were used. Next, the images were captured from each tissue section randomly, and a quantitative analysis was performed using ImageJ software (version 1.6.0_20, National Institutes of Health, Bethesda, MD, USA) with an RGB-based protocol as reported previously (Vrekoussis et al., 2009).

### Biochemical analysis

Blood samples were collected at 4 and 8 weeks, and biochemical analyses of serum levels of ammonia, aspartate transaminase, alanine aminotransferase, alkaline phosphatase, lactate dehydrogenase, total cholesterol (T-CHO) and TG were performed (Oriental Yeast Co., Shiga, Japan). Hydroxyproline in the liver was determined using a QuickZyme hydroxyproline kit (QuickZyme Biosciences, Leiden, The Netherlands).

### Expression of Tph1 in the small intestine

Total RNA was extracted from the small intestine using an RNeasy Mini kit (Qiagen, Hilden, Germany) and was reverse-transcribed into cDNA using a QuantiTect Reverse Transcription kit (Qiagen). Expression of tryptophan hydroxylase 1 (*Tph1*) and glyceraldehyde 3-phosphate dehydrogenase (*Gapdh*) was quantified by qRT-PCR using SYBR Green and the StepOnePlus System (Thermo Fisher Scientific, Waltham, MA, USA), and the results were analyzed with the bundled software. The following primers were used: *Tph1* (forward), 5′-ACCATGATTGAAGACAACAAGGAG-3′; *Tph1* (reverse), 5′-TCAACTGTTCTCGGCTGATG-3′; and *Gapdh* (QT01658692; Qiagen). The thermal conditions were as follows: 95°C for 10 min, followed by 40 cycles of 94°C for 15 s, 55°C for 30 s, and 72°C for 30 s, with the PCR product melting hold at 95°C for 15 s, 60°C for 1 min, and 95°C for 15 s. Changes in gene expression were evaluated using the 2−ΔΔCt method, with gene expression normalized to that of *Gapdh* in each sample.

### Analysis of the microbiota and SCFAs

Murine feces were collected from the cecum, and T-RFLP (Sjöberg et al., 2013) and SCFA analyses were performed by the Techno Suruga Laboratory (Shizuoka, Japan) according to previously reported methods (Takahashi et al., 2014; Nagashima et al., 2016). In detail, for T-RFLP, fecal samples (∼4 mg) were suspended in the solution [100 mM Tris-HCl (pH 9.0), 40 mM ethylenediaminetetraacetic acid (pH 8.0), 0.1 M guanidine thiocyanate, and 0.001% bromothymol blue)], and these samples were treated with zirconia beads using the FastPrep 24 Instrument (MP Biomedicals, Santa Ana, CA, USA). DNA was then extracted from the bead-treated suspensions according to the method described by Takahashi et al. (2014), and the final concentration of the DNA sample was adjusted to 10 ng/μl. Next, T-RFLP analysis for fecal samples was performed as described previously (Nagashima et al., 2016). The 16S rRNA gene was amplified from fecal DNA using a fluorescently labeled 516F primer (5′-TGCCAGCAGCCGCGGTA-3′; *E. coli* positions 516–532) and 1510R primer (5′-GGTTACCTTGTTACGACTT-3′; *E. coli* positions 1510–1492). The 5′ ends of the forward primers were labeled with 6-carboxyfluorescein, which was synthesized by Thermo Fisher Scientific. The PCR amplifications of DNA samples (10 ng of each DNA) were performed according to the protocol described by Nagashima et al., (2016). The purified PCR products (2 μl) were digested with 10 U of Fast Digest BslI (Thermo Fisher Scientific) at 37°C for 10 min. The T-RF fragment length was determined using an ABI PRISM 3130×l genetic analyzer (Thermo Fisher Scientific). The standard size marker was MapMarker X-Rhodamine Labeled 50 to 1000 bps (BioVentures, Little Rock, AR, USA). The T-RFs were divided into 31 operational taxonomic units (OTUs), which were quantified as the percentage of individual OTU per total OTU areas. The bacteria were predicted for each classification unit, and the corresponding OTU was identified according to the reference Fecal Microbiota T-RFLP Profiling (available in the public domain at www.tecsrg.co.jp/t-rflp/t_rflp_hito_OTU.html). The hierarchical clustering analysis of the individual OTUs based on Ward's method and principal component analysis were performed using the R-packages ‘amap’ and ‘ggplot’, respectively.

To determine SCFAs, 0.1 g feces was placed into a 2 ml tube with zirconia beads and suspended with 0.9 ml 0.5% phosphoric acid. The sample was heated at 85°C for 15 min, vortexed at 5 m/s for 45 s using FastPrep 24 (MP Biomedicals), and centrifuged at 16,873 ***g*** for 10 min. Then, 0.4 ml supernatant was transferred to a 1.5 ml tube, mixed with 0.4 ml ethyl acetate, shaken for 30 min and centrifuged at 16,873 ***g*** for 10 min. Then, 0.2 ml supernatant was mixed with 1 mM 4-methyl valeric acid as an internal standard. SCFAs in feces were then measured by gas chromatography with a flame ionization detector (7890B, Agilent Technologies, Santa Clara, CA, USA) and a capillary column DB-WAXetr (30 m, 0.25 mm id and 0.25 μm film thickness; Agilent Technologies). Helium was used as the carrier gas at 1.2 ml/min. The detector temperature was kept at 250°C. The oven temperature program was 50°C, then 10°C/min to 90°C, 15°C/min to 150°C, 5°C/min to 170°C, and 20°C to finally 250°C, and held for 4 min. Then, 1 µl extract was injected in the splitless mode.

### Statistical analyses

The data regarding time-dependent change of the BW and LW, histologic analyses, and serum biochemical factors were statistically evaluated by two-way repeated measure ANOVA or one-way ANOVA followed by Bonferroni's multiple comparison test or Student's *t*-test using GraphPad Prism7 software (version 7.04, MDF, Tokyo, Japan). *P*≤0.05 denoted statistical significance. For microbiota analysis, microbial diversity of OTUs within each group (α-diversity; observed OTU number and Shannon index) and across groups (β-diversity; Bray-Curtis index) were analyzed using R software (available in the public domain at www.r-project.org/). To compare similarities of the bacterial community structure between groups, the Wilcoxon rank-sum test was performed based on the observed OTU number, Shannon index and Bray–Curtis index using R software. Analysis of similarities and permutational analysis of variance were performed based on Euclidean distance calculated by R-packages ‘MASS’ and ‘vegan’. For all analyses, only *P*<0.05 was considered statistically significant.

## Supplementary Material

Supplementary information
