# Correction: Modulation of serotonin in the gut-liver neural axis ameliorates the fatty and fibrotic changes in non-alcoholic fatty liver

**DOI:** 10.1242/dmm.050622

**Published:** 2023-11-30

**Authors:** Masayoshi Ko, Kenya Kamimura, Takashi Owaki, Takuro Nagoya, Norihiro Sakai, Itsuo Nagayama, Yusuke Niwa, Osamu Shibata, Chiyumi Oda, Shinichi Morita, Atsushi Kimura, Ryosuke Inoue, Toru Setsu, Akira Sakamaki, Takeshi Yokoo, Shuji Terai

**Fig. 2B (original panel).**
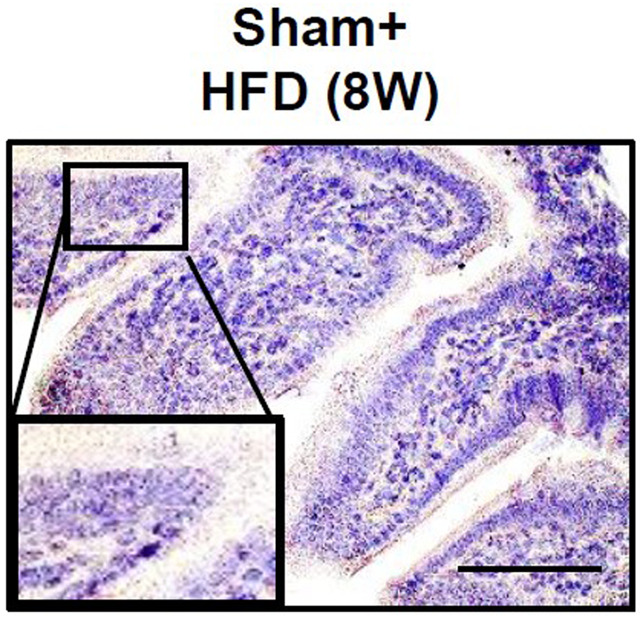
**Effect of autonomic neural signal transduction on the tight junction in the small intestine of NAFLD/NASH mice models.** (A,B) Representative images of Claudin-1 (A) and Zo-1 (B) staining of the small intestine of mice groups.

**Fig. 2B (corrected panel).**
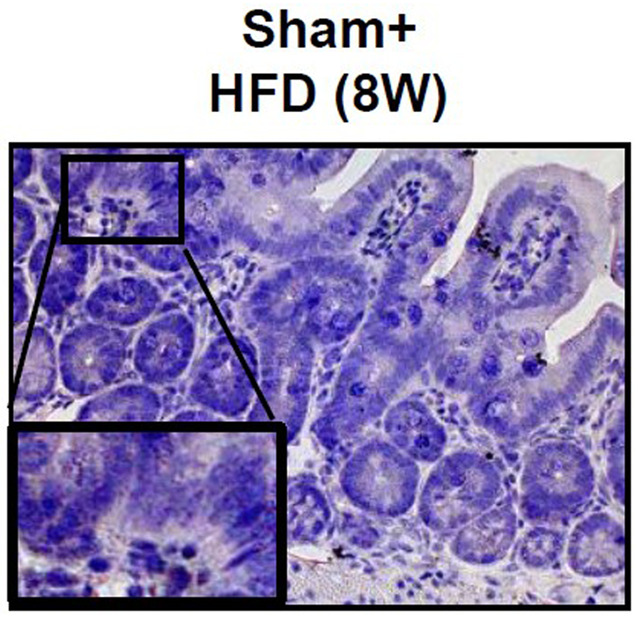
**Effect of autonomic neural signal transduction on the tight junction in the small intestine of NAFLD/NASH mice models.** (A,B) Representative images of Claudin-1 (A) and Zo-1 (B) staining of the small intestine of mice groups.

There was an error in *Dis. Model. Mech.* (2021) **14**, dmm048922 (doi:10.1242/dmm.048922).

An incorrect image was used for Sham+HFD (8W) in Fig. 2B. The original and corrected versions are shown below.

The online full text and PDF versions of the paper have been corrected. The authors apologise to readers for this error, which does not impact the results or conclusions of the paper.

