# Supplementary material for: Modulation of serotonin in the gut-liver neural axis ameliorates the fatty and fibrotic changes in non-alcoholic fatty liver
Source: Dis Model Mech. 2021 Mar 28;14(3):dmm048922. doi: 10.1242/dmm.048922 (PMC8084356; doi:10.1242/dmm.048922)
Supplement: Supplementary information [file dmm-14-048922-s1.pdf]

Figure S1

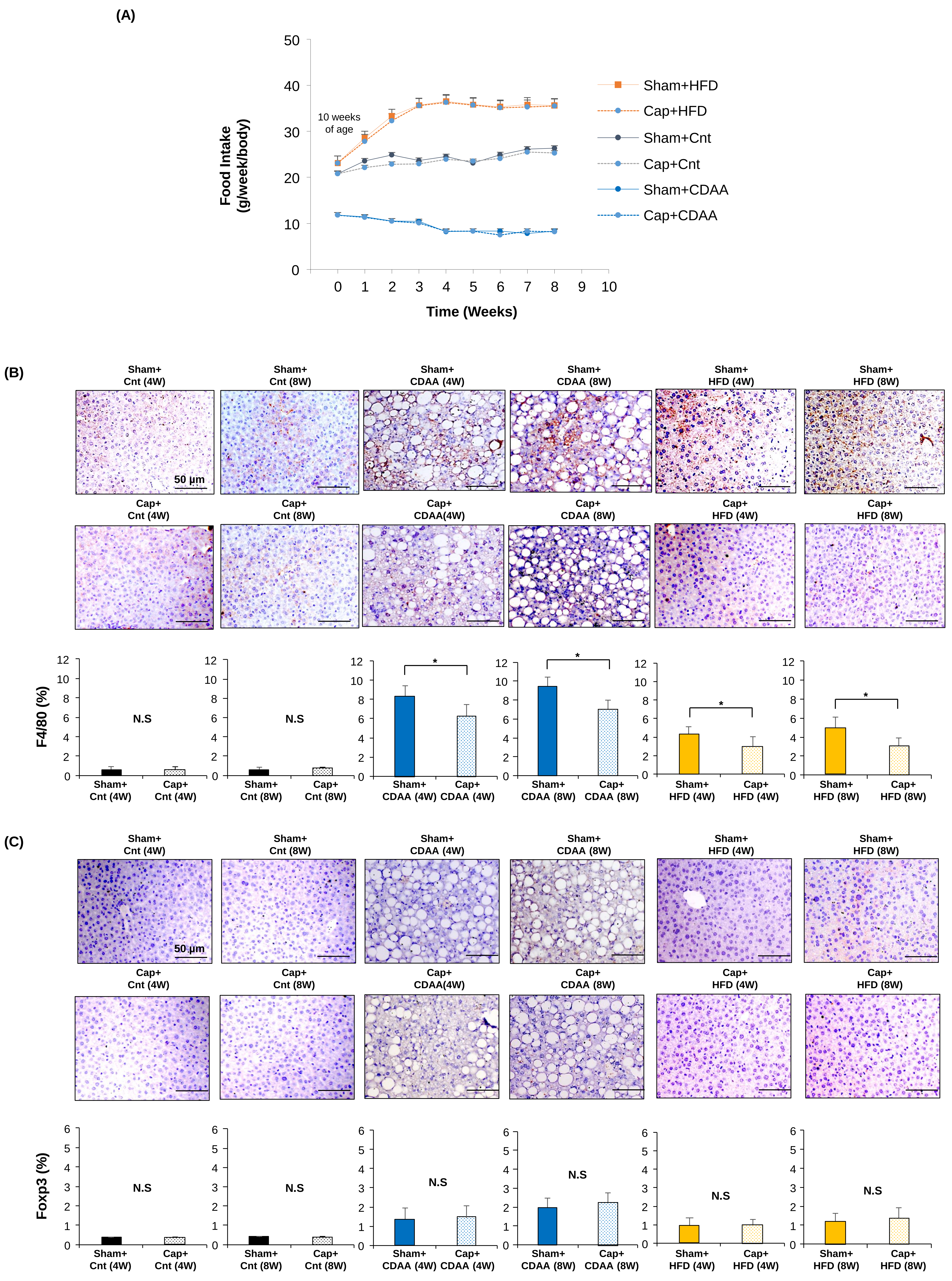

Disease Models & Mechanisms • Supplementary information

Fig. S1. Effect of autonomic neural signal transduction on inflammation in the liver of NAFLD/NASH mice models.

(A) Changes of food consumption. The values represent mean ± SD (*n* = 5 for each group).

(B) Representative images of F4/80 and Foxp3 (C) staining of the liver of mice groups. Scale bar represents 100 µm. Five different sections from each of the five mice (*n* = 25) in all groups were quantitatively analyzed for the positively stained area using ImageJ software. The values represent mean ± SD (*n* = 25 for each group), NS, no statistical significance. Student's *t*-test.

Disease Models & Mechanisms • Supplementary information

Figure S2

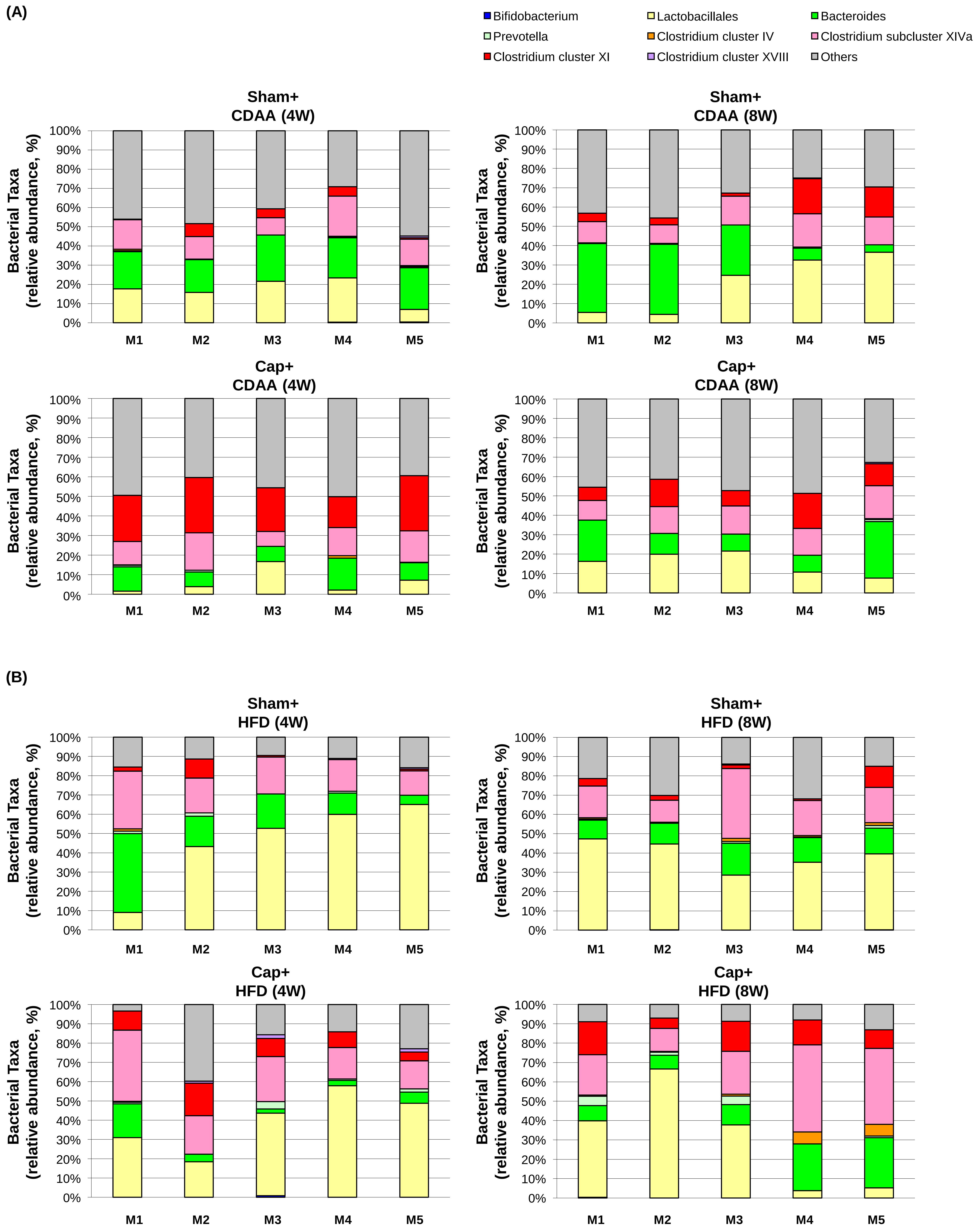

Disease Models & Mechanisms • Supplementary information

Fig. S2. Taxonomic distribution of microbiota. (A) Choline-deficient defined L-amino-acid (CDAA) diet-fed and (B) high-fat (HFD) diet-fed NAFLD model mice with or without autonomic nerve blockade. Values represent the average relative abundance across all samples within the indicated group (n=5, M1 represents Mouse #1).

Figure S3

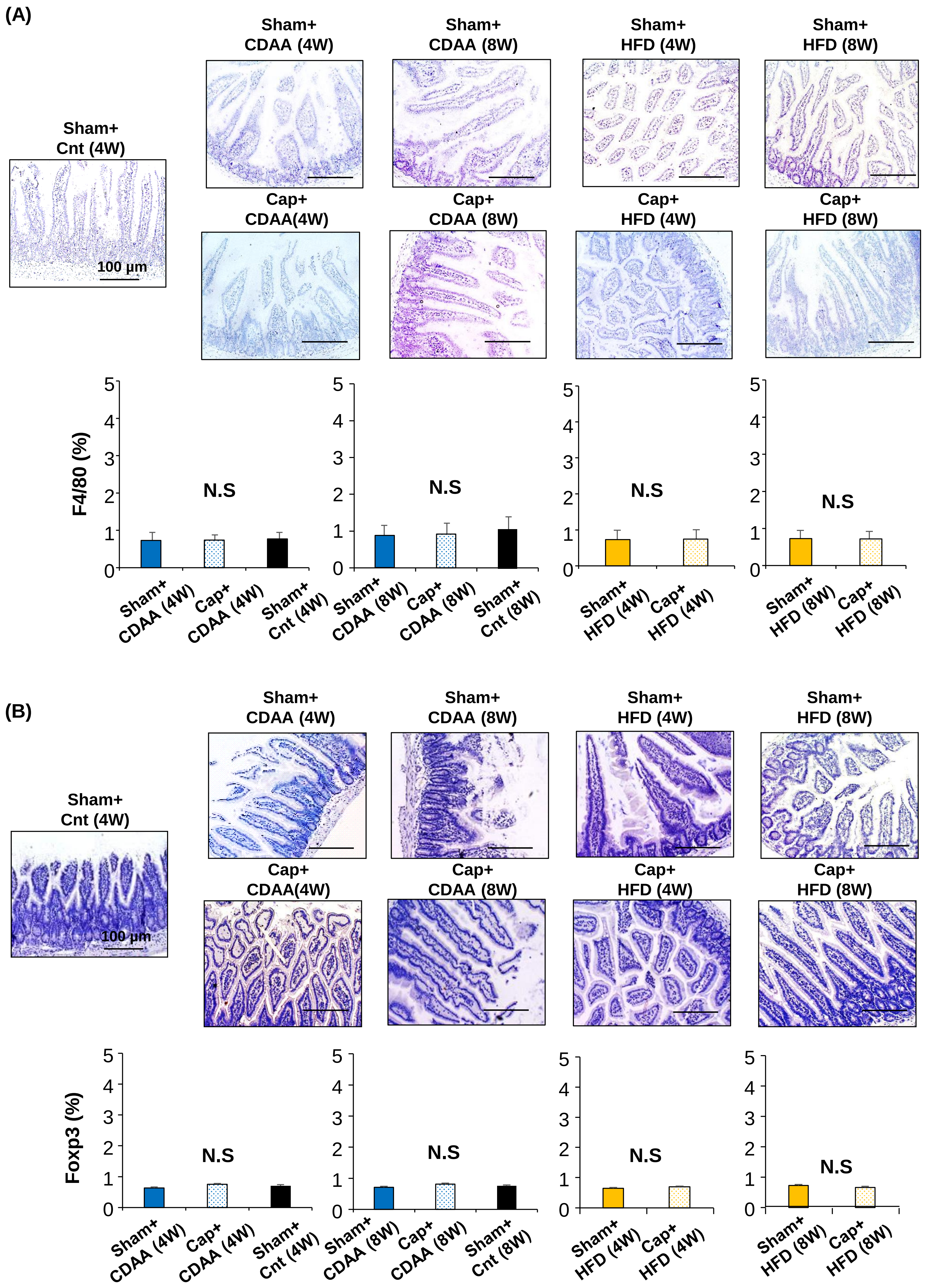

Disease Models & Mechanisms • Supplementary information

Fig. S3. Effect of autonomic neural signal transduction on small intestine inflammation of NAFLD/NASH mice models. Representative images of F4/80 (A) and Foxp3 (B) staining of the small intestine of mice groups. Scale bar represents 100 µm. Five different sections from each of the five mice (n = 25) in all groups were quantitatively analyzed for the positively stained area using ImageJ software. The values represent mean ± SD (n = 25 for each group), NS, no statistical significance. Student's t-test.
